# *AFF2* Is Associated With X-Linked Partial (Focal) Epilepsy With Antecedent Febrile Seizures

**DOI:** 10.3389/fnmol.2022.795840

**Published:** 2022-03-30

**Authors:** Dongfang Zou, Bing Qin, Jie Wang, Yiwu Shi, Peng Zhou, Yonghong Yi, Jianxiang Liao, Xinguo Lu

**Affiliations:** ^1^Epilepsy Center and Department of Neurology, Shenzhen Children’s Hospital, Shenzhen, China; ^2^Department of Neurology, Institute of Neuroscience, The Second Affiliated Hospital of Guangzhou Medical University, Guangzhou, China; ^3^Key Laboratory of Neurogenetics and Channelopathies of Guangdong Province and the Ministry of Education of China, Guangzhou, China; ^4^Epilepsy Center and Department of Neurosurgery, The First Affiliated Hospital of Jinan University, Guangzhou, China

**Keywords:** epilepsy, *AFF2* gene, whole-exome sequencing, intellectual disability, autism spectrum disorder

## Abstract

**Objective:**

*AFF2* mutations were associated with X-linked intellectual developmental disorder-109 and in males with autism spectrum disorder (ASD). The relationship between *AFF2* and epilepsy has not been defined.

**Method:**

Trios-based whole-exome sequencing was performed in a cohort of 372 unrelated cases (families) with partial (focal) epilepsy without acquired causes.

**Results:**

Five hemizygous missense *AFF2* mutations were identified in five males with partial epilepsy and antecedent febrile seizures without intellectual disability or other developmental abnormalities. The mutations did not present in the controls of general populations with an aggregate frequency significantly higher than that in the control populations. Previously, intellectual disability-associated *AFF2* mutations were genomic rearrangements and CCG repeat expansion mutations mostly, whereas the mutations associated with partial epilepsy were all missense. Missense *AFF2* mutations associated with epilepsy fell into the regions from N-terminal to the nuclear localization signal 1 (NLS1), while ASD-associated missense mutations fell in the regions from NLS1 to C-terminal.

**Conclusion:**

*AFF2* is potentially a candidate causative gene of X-link partial epilepsy with antecedent febrile seizures. The genotype–phenotype correlation and molecular sub-regional effect of *AFF2* help in explaining the mechanisms underlying phenotypic variations.

## Introduction

*AFF2* (OMIM* 300806) (also known as *FMR2* gene), which encodes AF4/FMR2 family member 2, is a transcriptional factor and RNA-binding protein that plays an important role in transcriptional regulation, RNA splicing, mRNA processing, and nuclear speckle organization (Bensaid et al., 2009). *AFF2* is highly conserved and abundantly expressed in human brain (Gecz et al., 1997), being essential for brain development. Homozygous *AFF2* knock-out mice showed abnormal central nervous system synaptic transmission, abnormal excitatory postsynaptic potential, and premature death.^1^ Previous studies have identified *AFF2* mutations in the etiology of X-linked intellectual developmental disorder-109 (MRX109), a form of mildly to moderately impaired intellectual development associated with learning difficulties, communication deficits, attention problems, hyperactivity, and autistic behavior (Knight et al., 1993, 1996; Mulley et al., 1995; Gecz, 2000), in which epileptic seizures were occasionally observed (Lo Nigro et al., 2000; Lesca et al., 2003). Several point mutations were also identified in patients with autism spectrum disorder (ASD) (Mondal et al., 2012; Jiang et al., 2013; Yuen et al., 2017). The relationship between *AFF2* and epilepsy has not been defined. In the present study, trios-based whole-exome sequencing (WES) was performed in a cohort of cases (families) of partial (focal) epilepsy without acquired causes. Five missense mutations of *AFF2* were identified in five unrelated individuals of partial epilepsy without intellectual disability or other developmental abnormalities.

## Materials and Methods

### Participants

A total of 372 cases (families) with partial epilepsy without acquired causes were recruited, including 323 cases from the Epilepsy Center of the Second Affiliated Hospital of Guangzhou Medical University in China between January 2013 and July 2020, and 49 cases from Shenzhen Children’s Hospital and the First Affiliated Hospital of Jinan University in China between January 2018 and July 2020. The probands included 230 males and 142 females and were subjected to trios-based WES for potential genetic etiology of epilepsy. The complete pedigree and clinical data of the probands were collected, including detailed clinical phenotypes, age of seizure onset, seizure type, seizure course and frequency, family history, treatment, prognosis, general and neurological examination, and brain magnetic resonance imaging (MRI). 24-h video electroencephalography (EEG) was performed on all patients, at the age ranging from 4 to 23 years (mean age, 10.2 years), and EEG was analyzed by at least two qualified electroencephalographers. The developmental and intellectual states of all patients (at the age range, 3–24 years; mean age, 10.4 years) were evaluated, including language, fine and gross motor, and adaptive social skills and performance at school or work. Gesell development scale, Wechsler intelligence scale for children-V, and Wechsler adult intelligence scale were utilized in the neuropsychological evaluation of the participants according to their ages. Seizure type and epilepsy syndrome were classified according to the criteria of the Commission on Classification and Terminology of International League of Against Epilepsy (ILAE) (No Author, 1985, 1989; Berg et al., 2010; Scheffer et al., 2017). The inclusion criteria were: (1) diagnosis of partial epilepsy based on ILAE criteria, characterized by focal seizures or focally originated focal to bilateral tonic-clonic seizures; (2) EEG examination presented focal discharges, including unilateral, bilateral, and multiple focal discharges with normal background. The exclusion criteria were: (1) diagnosis of generalized epilepsy based on ILAE criteria; (2) individuals with acquired causes, including brain tumors, head trauma, immune encephalitis, central nervous system infections, and cerebrovascular diseases.

This study was based on the guidelines of the International Committee of Medical Journal Editors concerning patient consent for research or participation. Written informed consent was obtained from all individuals or their legal guardians. The present study was approved by the Ethics Committee of the Second Affiliated Hospital of Guangzhou Medical University.

### Whole Exome Sequencing

Blood samples were obtained from all individuals and their parents. Genomic DNA was extracted from the peripheral blood using Qiagen Flexi Gene DNA kit (Qiagen, Hilden, Germany), according to the protocol of the manufacturer. Trio-based WES was conducted on the MGI 2000 platform by BGI-Shenzhen (Shenzhen, China) performing pair-end reads, 100 bp sequencing with 100–150 times average depth and more than 98% coverage of the target region. Deep sequencing data were aligned to the reference GRCh37 build (hg19) and variants were called according to the standard procedures as previously reported (Wang et al., 2018; Cai et al., 2019; Shi et al., 2019). We adopted a case-by-case analytical approach to identify candidate causative mutations in each trio. Firstly, we prioritized the rare variants with a minor allele frequency < 0.005 in the 1,000 Genomes Projects, Exome Aggregation Consortium, and Genome Aggregation Database (gnomAD). Secondly, we retained potentially pathogenic mutations, including frameshift, nonsense, canonical splice site, initiation codon, and missense mutations predicted as being damaging by 21 algorithms *in silico* prediction.^2^ Finally and importantly, potential disease-causing variants were screened under five models: (1) epilepsy-associated gene model; (2) *de novo* autosomal dominant model; (3) autosomal recessive inheritance model, including compound heterozygous and homozygous variants; (4) X-linked inheritance model; (5) co-segregation model. To identify novel epilepsy-associated genes, we put the known epilepsy-associated genes (Wang et al., 2017) aside. The genes with repetitively identified *de novo* variants, bi-allelic variants, hemizygous variants, and variants with segregations, were selected for further studies to define the gene-disease association. *AFF2* appeared as a candidate gene with recurrent hemizygous variants in this cohort of partial epilepsy and was subjected to further analysis in this study. The other potential candidate genes were not included in the present study. The candidate pathogenic variants were validated by Sanger sequencing. All variants in *AFF2* were annotated to the reference transcript NM_002025.4. Conservation of the mutated positions was evaluated by generating multiple sequence alignments of different species.

### Molecular and Genotype-Phenotype Correlation Analysis

To predict the effect of missense mutations on molecular structure, a protein model was established by Phyre2 (V 2.0).^3^ PyMOL 1.7 was used to visualize and analyze the three-dimensional protein structure and alteration of hydrogen bonds. Amino acids were annotated to the reference protein NP_002016. To evaluate the genotype-phenotype correlation, we reviewed all the relevant literature about *AFF2* mutations reported previously, including point mutations, genomic rearrangements, and CCG repeat expansion variants, as well as all the phenotypes of the mutations. All the mutations were retrieved from PubMed^4^ and Human Gene Mutation Database^5^ up to December 2020.

### Statistical Analysis

R statistical software (v3.4.1) and SPSS version 22.0 (SPSS, Inc., Chicago, IL, United States) were used for statistical analyses. Fisher’s exact test was applied to access the frequencies of *AFF2* mutations in the case cohort and the control populations. A *p-*value < 0.05 was considered statistically significant.

## Results

### Identification of *AFF2* Mutations

**FIGURE 1:**
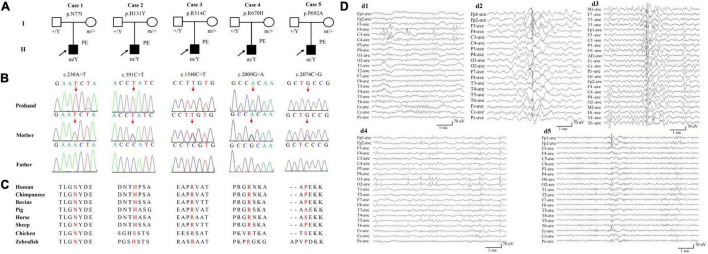
Genetic and electroencephalograms (EEG) of the cases with *AFF2* mutations. **(A)** Pedigrees of the five cases with *AFF2* mutations and their corresponding phenotypes. PE, partial epilepsy. **(B)** DNA sequence chromatogram of the *AFF2* mutations. Arrows indicate the positions of the mutations. **(C)** The amino acid sequence alignment of the five missense mutations shows that residues N77I, R514C, and R670H were highly conserved across various species. Residues H131Y and P692A were less conserved. **(D)** Changes of interictal EEG in the cases with *AFF2* mutations. **(d1)** Interictal EEG of case 1 showed bilateral frontal-central spike and slow waves (obtained at the age of 8 years). **(d2)** Interictal EEG of case 2 showed bilateral frontal and anterior-temporal spike and slow waves (at the age of 9 years). **(d3)** Interictal EEG of case 3 showed spike and slow waves predominant at left hemisphere (at the age of 5 years). **(d4)** Interictal EEG of case 4 showed sharp waves predominant at bilateral occipital regions (at the age of 3 years). **(d5)** Interictal EEG of case 5 showed spike and slow waves predominant at bilateral frontal regions (at the age of 17 years).

Five hemizygous missense *AFF2* variants were identified in five unrelated individuals with partial epilepsy, including c.230A > T/p.N77I, c.391C > T/p.H131Y, c.1540C > T/p.R514C, c.2009G > A/p.R670H, and c.2074C > G/p.P692A. All variants originated from their asymptomatic mothers (Figures 1A,B and Table 1). No *AFF2* variants were found in their fathers. The amino acid sequence alignment indicated that N77I, R514C, and R670H were located at residuals of highly conserved across various species (“highly conserved” means the fraction of the AFF2 residues conserved for the used species is 100%); while H131Y and P692A were located at residuals of less conserved according to the sequence alignment (“less conserved” means the fraction is less than 100%) (Figure 1C). None of the five cases was identified to have other pathogenic or likely pathogenic variants in the known genes associated with seizure disorders (Wang et al., 2017), neither other pathogenic nor likely pathogenic variants were found in the *de novo* autosomal dominant, autosomal recessive inheritance, and co-segregation model.

**TABLE 1:** Clinical features of the cases with *AFF2* mutations.

	Case 1	Case 2	Case 3	Case 4	Case 5
*AFF2* mutations (NM_002025.4)	c.230A > T/p.N77I	c.391C > T/p.H131Y	c.1540C > T/p.R514C	c.2009G > A/p.R670H	c.2074C > G/p.P692A
Diagnosis	PE	PE	PE	PE	PE
Gender	Male	Male	Male	Male	Male
Present age	8 yr	9 yr	5 yr	10 yr	27 yr
FS onset age	14 mo	2 yr	18 mo	10 mo	—
aFS onset age	7 yrs	9 yrs	5 yrs	7 yrs	8 yrs
Intermission from FS to EP	6 yrs	7 yrs	4 yrs	7 yrs	—
Seizure type	FS, CPS	FS, CPS	FS, CPS	FS, CPS, sGTCS	CPS, sGTCS
Seizure frequency	FS once, CPS 2 times/mo	FS once, CPS 3 times/mo	FS 4 times, CPS 4–5 times/yr	Prolonged FS 2 times/yr, CPS/sGTCS 3–4 times/yr	CPS 5–6 times/mo, sGTCS 1–2 times/yr
Seizure timing	Nocturnal	Diurnal	Diurnal	Diurnal and nocturnal	Diurnal and nocturnal
Family history of seizure	None	None	None	None	None
Ictal EEG	Generalized spike and spike-slow wave originated from the left mid-central with a partial seizure	NA	Generalized slow wave originated from the left hemisphere with a partial seizure	NA	NA
Interictal EEG	Bilateral spike and slow waves predominantly in the frontal and central areas	Bilateral spike and slow waves predominantly in the frontal and anterior-temporal areas	Spike and slow waves predominantly in the left hemisphere	Bilateral sharp waves predominantly in the occipital areas	Bilateral spike and slow waves predominantly in the frontal areas
Brain MRI	Normal	Normal	Normal	Normal	Normal
Intelligence	Normal	Normal	Normal	Normal	Normal
Developmental delay	No	No	No	No	No
Treatment	LTG	VPA, OXC	LTG	VPA, OXC	VPA, LTG
Seizure outcome	Free for 1 yr	Free for 1 yr	Free for 1 yr	Free for 1 yr	Free for 5 yrs

*aFS, afebrile seizures; CPS, complex partial seizure; EEG, electroencephalogram; EP, epilepsy; FS, febrile seizures; LTG, lamotrigine; MRI, magnetic resonance imaging; mo, month; NA, not available; OXC, oxcarbazepine; PE, partial epilepsy; sGTCS, secondary generalized tonic-clonic seizure; VPA, valproate; yr, year.*

We compared the aggregate frequency of the five hemizygous *AFF2* mutations in this cohort with that in the populations in gnomAD, or controls of gnomAD, according to the aggregate variant analysis method suggested by ClinGen (Strande et al., 2017). Five mutant alleles in a total of 230 alleles (230 males in the 372 cases) were detected in this cohort. Mutation R514C had an extremely low frequency (9.95e-06) but was not present in the controls of gnomAD-East Asian and gnomAD-all population, part of the allele frequency was from the 1000G data. The other four mutations in *AFF2* had no allele frequency in gnomAD databases (Table 2). The differences in aggregate frequencies of the mutant alleles between this cohort in male cases and the male controls in gnomAD (general population, East-Asian population) were statistically significant (5/230 vs. 0/46329 in the male controls of gnomAD-all population, *p* = 2.82 × 10^–12^; 5/230 vs. 0/3058 in the male controls of gnomAD-East Asian population, *p* = 1.61 × 10^–6^, respectively) (Table 2).

**TABLE 2:** Analysis of the aggregate frequency of *AFF2* mutations identified in this study.

	Allele count/number in this study (%)	Allele count/number in male controls of gnomAD-all populations (%)	Allele count/number in male controls of gnomAD-East Asian populations (%)
**Identified *AFF2* mutations**			
chrX:147743478 (c.230A > T/p.N77I)	1/230 (0.43)	-/-	-/-
chrX:147743639(c.391C > T/p.H131Y)	1/230 (0.43)	-/-	-/-
chrX:148035252(c.1540C > T/p.R514C)	1/230 (0.43)	-/-	-/-
chrX:148037584(c.2009G > A/p.R670H)	1/230 (0.43)	-/-	-/-
chrX:148037649(c.2074C > G/p.P692A)	1/230 (0.43)	-/-	-/-
**Total**	5/230 (2.17)	0/46329 (0)	0/3058 (0)
***p-*value**		2.82 × 10^–12^	1.61 × 10^–6^
**OR (95% CI)**		Inf (186.76–Inf)	Inf (12.33–Inf)

*p-values and odds ratio were estimated with two-sided Fisher’s exact test.*

*CI, confidence interval; gnomAD, Genome Aggregation Database; OR, odds ratio.*

*Ref: Cai et al. (2015).*

### Clinical Features of the Cases With *AFF2* Mutations

*AFF2* mutations were identified in five unrelated male epilepsy patients with focal seizures and/or focal discharges, ranging in age from 5 to 27 years. The clinical features of the five cases were summarized in Table 1. The age of febrile seizures onset ranged from 10 months to 2 years, and the afebrile seizures started from 5 to 9 years. All the cases presented with infrequent focal impaired awareness seizures, and the cases with mutation R670H (Case 4) and P692A (Case 5) also had focal to bilateral tonic-clonic seizures. Except for the case with mutation P692A (Case 5), the other four cases exhibited antecedent febrile seizures or prolonged febrile seizures, and they developed afebrile seizures later with an intermission of 4–7 years. The interictal EEG recording showed epileptiform discharges, including bilateral, unilateral, and multiple discharges, predominantly at frontal, central, and temporal lobe, predominantly during sleeping. Trends of generalization were also observed in the case with mutation R514C (Case 3) (Figure 1D). The ictal EEG of the case with mutation N77I (Case 1) showed an onset originated from the left mid-central area, and the case with mutation R514C (Case 3) demonstrated a seizure originated from the left hemisphere. Brain MRIs of all the cases were normal. According to the results of the relevant developmental scales, all individuals presented normal development and intellectual ability. The epilepsy of the five cases was controlled with one or two antiepileptic drugs. The cases with mutation N77I (Case 1) and R514C (Case 3) achieved seizure free with monotherapy of lamotrigine (6–7 mg/kg/d). The cases with H131Y (Case 2) and R670H (Case 4) had seizure reductions with valproate (20–25 mg/kg/d) at first and became seizure free after the add-on of oxcarbazepine (20–25 mg/kg/d). The case with P692A (Case 5) had seizure reduction of more than 60% with initial valproate (20 mg/kg/d) and got seizures controlled on the combined use of lamotrigine (5 mg/kg/d).

### Molecular Effect of *AFF2* Mutations

**FIGURE 2:**
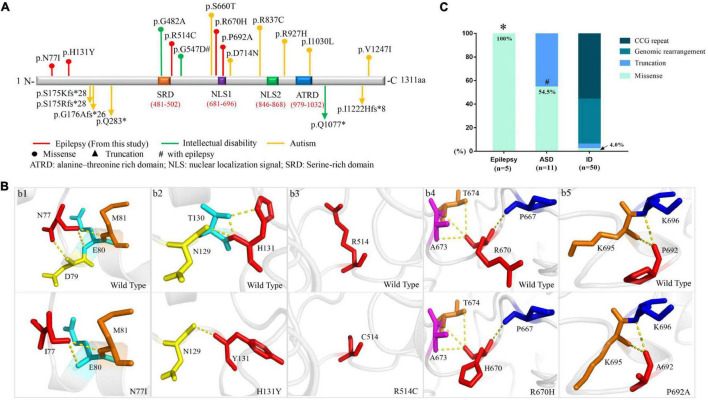
Schematic presentation of FMR2 structure and genotype-phenotype correlation of *AFF2*. **(A)** Schematic diagram of missense and destructive *AFF2* mutations and their locations on FMR2 protein. Missense mutations were shown at the top of the structural diagram. Destructive mutations were shown at the bottom. **(B)** Schematic illustration of the changes in hydrogen bonds. The residues where the mutations occurred are shown as red rods. The hydrogen bonds are shown as yellow spheres. **(C)** Genotypes of *AFF2* in epilepsy, autism spectrum disorder (ASD), and intellectual disability (ID). *The proportion of missense mutations in epilepsy is significantly higher than that in ID (*p* < 0.001) through Fisher’s exact test. ^#^The proportion of missense mutations in ASD is significantly higher than that in ID (*p* < 0.001) through Fisher’s exact test.

The length of amino acid of FMR2 is 1311, including two bipartite NLSs (nuclear localization signals)–NLS1 (RKEPRPNIPLAPEKKK) at amino acid position 681–696 and NLS2 (KPAPKGKRKHKPIEVAEKIPEKK) at position 846–868. Additionally, a large serine-rich domain locates at protein position 481–502, which overlaps with the regions involved in transcription activation, and a large alanine-threonine-rich domain is at position 979–1032, being unique to the FMR2 (Gecz et al., 1997). N-terminal domain of the protein includes amino acids 1–541, which is known to have transactivation activity (Hillman and Gecz, 2001), whereas C-terminal domain comprises amino acids 633–1272, being responsible for localization of the protein in nuclear speckles (Bensaid et al., 2009). In the present study, mutations N77I, H131Y, and R514C were located in the N-terminal domain. P692A was located in the NLS1 domain, while R670H was located in the N-terminal frank of NLS1 (Figure 2A).

The molecular effect of the five missense mutations was analyzed by protein modeling using PHYRE2^6^ and PyMOL with the templates available (Figure 2B). The protein modeling showed that mutations N77I and H131Y resulted in alteration of hydrogen bonds and potentially affected the protein steric configuration. Originally, residue N77 formed three hydrogen bonds with residue D79, E80, and M81, respectively. When asparagine was replaced by isoleucine at residue N77, the hydrogen bond with D79 was destroyed (Figure 2Bb1). Residue H131 formed two hydrogen bonds with residue T130 and one hydrogen bond with residue N129. When histidine was replaced by tyrosine at residue H131, the hydrogen bonds with residue T130 were destroyed (Figure 2Bb2). There was no hydrogen bond formed with other surrounding residues at residue C514 (Figure 2Bb3). Mutation R670H and P692A showed no hydrogen bond altered at the residues (Figures 2Bb4,b5).

### Genotype-Phenotype Correlation and Molecular Sub-Regional Implication

We reviewed the previous studies through PubMed and Human Gene Mutation Database and analyzed the relationship between genotype and phenotype of *AFF2* mutations. Previously, 33 *AFF2* mutations that were associated with diseases of the central nervous system have been reported (Supplementary Table 1), including 8 missense mutations, 6 destructive (null) mutations (2 nonsense, 1 small deletion, and 3 small insertions), and 19 genomic rearrangements (15 gross deletions, 3 gross insertions, and 1 complex rearrangement). Additionally, 28 cases/families with CCG repeat expansion (≥ 200 repeats or > 5.2 Kb) in 5′-UTR have been published. The missense mutations were identified in 6 cases with ASD (6/8) and 2 cases with intellectual disability (2/8, one of whom accompanied with epilepsy). The destructive mutations were associated with ASD (5/6) mostly, and a case with intellectual disability (1/6). The genomic rearrangements were all associated with intellectual disability, among which 15 cases accompanied with developmental delay (15/19), 9 cases with epilepsy (9/19), and 1 case with ASD (1/19). Similarly, the CCG repeat variants were all associated with intellectual disability (Figure 2C and Supplementary Table 1).

In this study, the 5 cases who presented partial epilepsy all harbored missense mutations (5/5, 100%). In contrast, among the patients with ASD, six cases harbored missense mutations (6/11, 54.5%) and five cases harbored truncated mutations (5/11, 45.5%). In the patients with intellectual disability, missense mutations accounted for 4.0% (2/50), truncating mutation accounted for 2.0% (1/50), genomic rearrangements accounted for 38.0% (19/50), and CCG repeat variants accounted for 56.0% (28/50) of the cases. The proportion of missense mutations in epilepsy without intellectual disability and ASD was 100%, which was significantly higher than that in intellectual disability (*p* < 0.001) (Figure 2C).

Our previous studies showed that the molecular sub-regional location of the missense mutations was associated with the phenotypic variation and considered to be a critical factor to determine the pathogenicity of variants (Liu et al., 2020; Tang et al., 2020). We thus analyzed the molecular sub-regional implications of *AFF2* variants on phenotype variation. The missense mutations with epilepsy in the present study fell into the regions from the N-terminal to the NLS1, whereas the missense mutations with ASD fell in the regions from the NLS1 to the C-terminal, with several mutations overlapped around the NLS1 (Figure 2A).

## Discussion

*AFF2* is a large gene containing 22 exons and spanning about 500 kb that is located on chromosome Xq28. FMR2, the *AFF2* encoded protein, has five annotated isoforms. The longest one is composed of 1,311 amino acids and contains two nuclear localization signals (Gecz et al., 1997). FMR2 acts as a transcriptional factor and RNA-binding protein, playing an essential role for transcriptional regulation and RNA splicing in nuclear speckle (Bensaid et al., 2009). In the present study, five novel missense *AFF2* mutations were identified in five male individuals with partial epilepsy and antecedent febrile seizures. The mutations were inherited from their asymptomatic mothers, consistent with an X-linked inheritance pattern. This study suggests that *AFF2* is potentially a candidate causative gene of X-link partial epilepsy with antecedent febrile seizures. The molecular sub-regional effect of *AFF2* helps in explaining the mechanisms underlying phenotypic variations.

Homozygous knock-out of *Aff2* in the mice model resulted in premature death with incomplete penetrance. The survival mice showed impaired learning and memory abilities and increased long-term potentiation—similar features as human intellectual disability (Gu et al., 2002), supporting the view that FMR2 is responsible for intellectual disability. Previous studies showed that intellectual disability associated *AFF2* mutations were genomic rearrangements and CCG repeat expansion mostly (Figure 2C and Supplementary Table 1), suggesting a pathogenic role of loss-of-function of *AFF2* in intellectual disability. Epileptic seizures were also observed in patients with genomic rearrangements (Wolff et al., 1997; Moore et al., 1999), suggesting that loss-of-function of *AFF2* would be potentially associated with epilepsy. In the present study, the patients presented good responses to antiepileptic drugs and become seizure free. These findings suggested that missense *AFF2* mutations were potentially associated with epilepsy with favorable outcomes without intellectual disability.

Apart from epilepsy, missense *AFF2* mutations have also been identified in patients with ASD (Mondal et al., 2012; Jiang et al., 2013). Further analysis revealed that missense *AFF2* mutations with epilepsy in this study mainly fell into the regions from the N-terminal to the NLS1, whereas the missense mutations with ASD mainly fell in the regions from the NLS1 to the C-terminal (Figure 2A). This evidence suggested a molecular sub-regional effect of *AFF2* mutations, as that in several genes reported previously (Liu et al., 2020; Tang et al., 2020). FMR2 is a multifunctional protein connecting transcriptional regulation and RNA splicing and plays an important role in regulating gene expression in the cell nucleus (Gecz, 2000). The protein shuttles between speckles and splicing sites, where it can pick up cargo RNAs and transfer them to the nucleolus for subsequent modifications (Bensaid et al., 2009). A previous study showed that FMR2 acted as a potent transcription activator and could regulate transcription via its N-terminal region (Hillman and Gecz, 2001). On the other hand, FMR2 protein is also an RNA binding protein, co-localizing with the splicing factor SC35 in nuclear speckles and is involved in splicing of *FMR1* pre-mRNA through its specific interaction with G-quadruplex RNA-forming structure via its C-terminal domain (Lamond and Spector, 2003). The G-quadruplex structure was known to work as an exonic splicing enhancer which could control splicing efficiency (Bensaid et al., 2009). Therefore, the N-terminal region of FMR2 was closely associated with transcription and C-terminal region was able to modulate splicing, providing a molecular basis of the sub-regional effect of *AFF2* mutations and potentially explaining the underlying mechanisms of phenotypic variations.

In this study, the cases all presented with partial epilepsy. *AFF2* is ubiquitously expressed in multiple human brain tissues (Gecz et al., 1997) and is more abundant in the frontal cortex, anterior cingulate cortex, hippocampus, and the amygdala, which may provide an anatomical basis for the phenotype of partial epilepsy. Moreover, four cases presented with antecedent febrile seizures at the average age of 16.5 months (from 10 months to 2 years old). The intermission from febrile seizures to epilepsy was 4–7 years. It is notable that *AFF2* is significantly expressed in the fetal and adult brain (Gecz et al., 1997). The data from Unigene database in NCBI showed that the expression of *AFF2* was high in blastocyte and fetus, with an intermission in early life, and then increased again in adults (Supplementary Figure 1). The intermission of gene expression was consistent with the intermission between febrile seizures and epilepsy presented in the patients. The relationship between gene expression and occurrence of phenotype potentially implies clinical significance in evaluation of the course and outcome of the illness.

There were several limitations in this study. First, the functional consequences of these missense variants warrant further validation by experimental studies. Second, previous studies revealed that some of the *AFF2* mutations associated with intellectual disability were CCG repeat expansion, which were not included in the present study. Third, a previous study reported that a missense mutation of *AFF2* was identified in a 6-year-old boy with focal epilepsy and moderate intellectual disability, but without febrile seizures (Zhang et al., 2015). The study indicated that missense *AFF2* mutations could also lead to other phenotypes such as intellectual disability, possibly associated with the molecular sub-regional location. Further studies are required to verify the association. Last, the sample size was limited with geographical limitations. Larger cohorts are required for validation by multicenter research.

## Conclusion

This study identified five missense *AFF2* mutations in five unrelated males with partial epilepsy and antecedent febrile seizures without intellectual disability or other developmental abnormalities. The frequency of the identified mutant alleles in this cohort was significantly higher than that in the control populations in gnomAD. Further analysis revealed that the *AFF2* mutations associated with partial epilepsy in this study were all missense, in contrast, intellectual disability-associated mutations were genomic rearrangements and CCG repeat expansion mostly. These findings suggested *AFF2* was potentially a candidate causative gene of X-link partial epilepsy with antecedent febrile seizures. The genotype-phenotype correlation and molecular sub-regional effect of *AFF2* help in explaining the mechanisms underlying phenotypic variations.

## Data Availability Statement

The datasets presented in this study can be found in online repositories. The names of the repository/repositories and accession number(s) are the following: Genbank (https://www.ncbi.nlm.nih.gov/nuccore/) with accession numbers: GenBank MZ958719–MZ958733.

## Ethics Statement

The studies involving human participants were reviewed and approved by the Ethics Committee of the Second Affiliated Hospital of Guangzhou Medical University. Written informed consent to participate in this study was provided by the participants’ legal guardian/next of kin.

## Author Contributions

XL designed the study, administered the project, and revised the manuscript. JL designed the study and contributed to data interpretation. DZ completed collection of the clinical data, analysis of the data, and draft of the manuscript. XL and DZ completed the recruitment of the patients and contributed to data interpretation. BQ, JL, and YY contributed to data analysis and interpretation. JW, PZ, and YS performed data analysis and provided technical assistance. All authors have read and approved the final manuscript.

## Conflict of Interest

The authors declare that the research was conducted in the absence of any commercial or financial relationships that could be construed as a potential conflict of interest.

## Publisher’s Note

All claims expressed in this article are solely those of the authors and do not necessarily represent those of their affiliated organizations, or those of the publisher, the editors and the reviewers. Any product that may be evaluated in this article, or claim that may be made by its manufacturer, is not guaranteed or endorsed by the publisher.

## Footnotes

1
http://www.informatics.jax.org/allele/MGI:2182641

2
http://varcards.biols.ac.cn/

3
http://www.sbg.bio.ic.ac.uk/~phyre2/html/page.cgi?id=index

4
https://pubmed.ncbi.nlm.nih.gov/

5
http://www.hgmd.cf.ac.uk/ac/index.php

6
http://www.sbg.bio.ic.ac.uk/p̃hyre2/html/page.cgi?id=index
