# Supplementary material for: AFF2 Is Associated With X-Linked Partial (Focal) Epilepsy With Antecedent Febrile Seizures
Source: Front Mol Neurosci. 2022 Mar 30;15:795840. doi: 10.3389/fnmol.2022.795840 (PMC9006616; doi:10.3389/fnmol.2022.795840)
Supplement: Supplementary file 1 [file Data_Sheet_1.pdf]

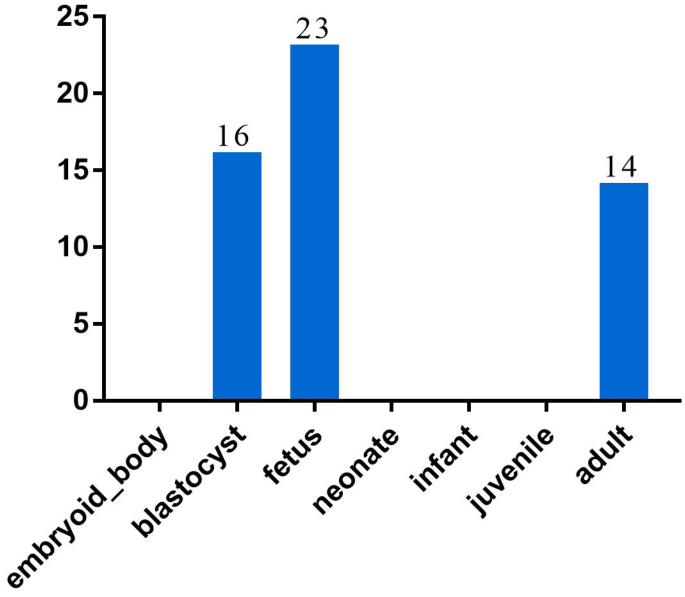

Supplementary Figure 1. Gene expression level in different stages. The data was from Unigene database in NCBI.

Supplementary Table 1. Summary of genetic and phenotypic features of the cases with *AFF2* mutations

Mutation type	Mutation	Inheritance	Exon	Age of seizure onset	Sex	ID	GDD	ASD	EP	Reference
**Point variants**										
**Missense**	c.230A>T/p.N77I	maternal	3	14mo	M	−	−	−	+	This study
	c.391C>T/p.H131Y	maternal	3	2yr	M	−	−	−	+	This study
	c.1445G>C/p.G482A	maternal	10	−	M	+	−	−	−	Farwell (2015)(Farwell et al., 2015)
	c.1540C>T/p.R514C	maternal	10	18mo	M	−	−	−	+	This study
	c.1640G>A/p.G547D	maternal	11	6yr	M	+	−	−	+	Zhang (2015)(Zhang et al., 2015)
	c.1979G>C/p.S660T	maternal	11	−	M	−	−	+	−	Mondal (2012)(Mondal et al., 2012)
	c.2009G>A/p.R670H	maternal	11	10mo	M	−	−	−	+	This study
	c.2074C>G/p.P692A	maternal	11	8yr	M	−	−	−	+	This study
	c.2140G>A/p.D714N	maternal	11	−	M	−	−	+	−	Mondal (2012)(Mondal et al., 2012)
	c.2509C>T/p.R837C	maternal	11	−	M	−	−	+	−	Mondal (2012)(Mondal et al., 2012)

	c.2780G>A/p.R927H	maternal	13	—	M	—	—	+	—	Mondal (2012)(Mondal et al., 2012)
	c.3088A>C/p.I1030L	*De novo*	14	—	M	—	—	+	—	Mondal (2012)(Mondal et al., 2012)
	c.3739G>A/p.V1247I	maternal	20	—	M	—	—	+	—	Jiang (2013)(Jiang et al., 2013)
**Nonsense**	c.847C>T/p.Q283*	NA	3	—	M	—	—	+	—	Lim (2013)(Lim et al., 2013)
	c.3229C>T/p.Q1077*	NA	15	—	M	+	—	—	—	Grozeva (2015)(Grozeva et al., 2015)
**Small deletions**	c.527_528delGT/p.G176Afs*26	maternal	3	—	M	—	—	+	—	Yuen (2017)(RK et al., 2017)
**Small insertions**	c.523dupA/p.S175Kfs*28	maternal	3	—	M	—	—	+	—	Yuen (2017)(RK et al., 2017)
	c.524_525insA/S175Rfs*28	maternal	3	—	M	—	—	+	—	Yuen (2017)(RK et al., 2017)
	c.3663dupC/Ile1222Hisfs*8	maternal	20	—	M	—	—	+	—	Kosmicki (2017)(Kosmicki et al., 2017)
**Genomic rearrangement**										
**Gross deletions**	chrX:147.21_148.54	*De novo*	—	—	M	+	+	—	—	Willemsen (2012)(Willemsen et al., 2012)

	ChrX:147,462,961-147,478,060_147,599,458-147,608,722	maternal	3	−	M	+	−	+	−	Stettner (2011)(Stettner et al., 2011)
	chrX:147,478,059_147,718,495	maternal	2-4	−	M	+	+	−	−	Sahoo (2011)(Sahoo et al., 2011)
	chrX:147,043,944_147,543,272	NA	1-3	−	M	+	+	−	−	Sahoo (2011)(Sahoo et al., 2011)
	chrX: 147.7–148.6 Mb	NA	7-11	−	M	+	+	−	−	Honda (2007)(Honda et al., 2007)
	chrX:139,990,405–149,404,134	maternal	Whole gene	NA	M	+	+	−	+	Brusius-Facchin (2012)(Brusius-Facchin et al., 2012)
	chrX:144,726,761–148,623,869	*De novo*	Whole gene	NA	M	+	+	−	+	Brusius-Facchin (2012)(Brusius-Facchin et al., 2012)
	DXS7536 proximally to *FMR2* distally	*De novo*	Whole gene	NA	M	+	+	−	+	Moore (1999)(Moore et al., 1999)
	DXS984 proximally to DXS1193 distally	maternal	Whole gene	5yr	M	+	+	−	+	Wolff (1997)(Wolff et al., 1997)

	DXS984 distally to *FMR2* distally	*De novo*	Whole gene	18mo	F	+	+	−	+	Wolff (1997)(Wolff et al., 1997)
	DXS312 proximally to DXS1193 distally	*De novo*	Whole gene	25mo	M	+	+	−	+	Albright (1994)(Albright et al., 1994)
	IDS up to part of *FMR2*	NA	NA	NA	M	+	+	−	+	Timms (1997)(Timms et al., 1997)
	IDS up to *FMR2*	NA	Whole gene	NA	M	+	+	−	+	Timms (1997)(Timms et al., 1997)
	Out-of-frame deletion including exon 2 and 3	NA	2, 3	−	M	+	+	−	−	Gedeon (1995)(Gedeon et al., 1995)
	<100 kb deletion, part of *FMR2*	NA	NA	−	M	+	−	−	−	Gedeon (1995)(Gedeon et al., 1995)
**Gross insertions**	chrX:140,033,727_151,588,281	*De novo*	Whole gene	−	M	+	+	−	−	Isrie (2012)(Isrie et al., 2012)
	chrX:147,646,015_ 147,837,382	NA	Whole gene	NA	M	+	−	−	+	Isrie (2012)(Isrie et al., 2012)
	chrX:147547319_147757141	NA	3-7	−	M	+	−	−	−	Whibley (2010)(Whibley et al., 2010)
**Complex**	46,X,t(X;15)(q28;p11.2)	*De novo*	−	−	F	+	+	−	−	Honda

**rearrangement**										(2007)(Honda et al., 2007)
**CCG repeat variations**										
	(CCG)n, Δ＞650bp (2 families)	maternal	－	－	M	＋	－	－	－	Knight (1993)(Knight et al., 1993)
	(CCG)n, Δ≥0.5Kb (6 families)	maternal	－	－	M/F	＋	－	－	－	Mulley (1995)(Mulley et al., 1995)
	(CCG)n, Δ≥1.6Kb (1 family)	maternal	－	－	M	＋	－	－	－	Carbonell (1996)(Carbonell et al., 1996)
	(CCG)n, ≥200 repeats (4 family)	maternal	－	－	M	＋	－	－	－	Biancalana (1996)(Biancalana et al., 1996)
	(CCG)n, Δ≥1.0Kb (4 cases)	maternal	－	－	M	＋	－	－	－	Knight (1996)(Knight et al., 1996)
	(CCG)n, Δ≥0.3Kb (1 family)	maternal	－	－	M	＋	－	－	－	Mila (1997)(Mila et al., 1997)
	(CCG)n, ＞5.2Kb (2 families)	maternal	－	－	M	＋	－	－	－	Abrams (1997)(Abrams et al., 1997)
	(CCG)n, Δ≥1.4Kb (3 families)	maternal	－	－	M	＋	－	－	－	Barnicoat (1997)(Barnicoat et al., 1997)

(CCG)n, Δ≥1.0Kb (3 families)	maternal	－	－	M	＋	－	－	－	Gecz (1997)(Gecz et al., 1997)
(CCG)n, Δ＞400bp (1 case)	maternal	－	NA	M	＋	－	－	＋	Lo Nigro (2000)(Lo Nigro et al., 2000)
(CCG)n, ≥600 repeats (1 family)	maternal	－	NA	M/F	＋	－	－	－/＋	Lesca (2003)(Lesca et al., 2003)

Abbreviations: ASD: autism spectrum disorder; GDD: global developmental delay; Ep: epilepsy; F: female; M: male; ID: intellectual disability; NA: not available; Δ: the size of the increase above the baseline of CCG expansion fragments 5.2 kb.

References

Abrams, M. T., Doheny, K. F., Mazzocco, M. M., Knight, S. J., Baumgardner, T. L., Freund, L. S., et al. (1997). Cognitive, behavioral, and neuroanatomical assessment of two unrelated male children expressing FRAXE. Am J Med Genet. 74, 73-81. doi:10.1002/(sici)1096-8628(19970221)74:1<73::aid-ajmg16>3.0.co;2-o

Albright, S. G., Lachiewicz, A. M., Tarleton, J. C., Rao, K. W., Schwartz, C. E., Richie, R., et al. (1994). Fragile X phenotype in a patient with a large de novo deletion in Xq27-q28. Am J Med Genet. 51, 294-297. doi:10.1002/ajmg.1320510403

Barnicoat, A. J., Wang, Q., Turk, J., Green, E., Mathew, C. G., Flynn, G., et al. (1997). Clinical, cytogenetic, and molecular analysis of three families with FRAXE. J Med Genet. 34, 13-17. doi:10.1136/jmg.34.1.13

Biancalana, V., Taine, L., Bouix, J. C., Finck, S., Chauvin, A., De Verneuil, H., et al. (1996). Expansion and methylation status at FRAXE can be detected on EcoRI blots used for FRAXA diagnosis: analysis of four FRAXE families with mild mental retardation in males. Am J Hum Genet. 59, 847-854.

Brusius-Facchin, A. C., De Souza, C. F., Schwartz, I. V., Riegel, M., Melaragno, M. I., Correia, P., et al. (2012). Severe phenotype in MPS II patients associated with a large deletion including contiguous genes. Am J Med Genet A. 158A, 1055-1059. doi:10.1002/ajmg.a.35271

Carbonell, P., Lopez, I., Gabarron, J., Bernabe, M. J., Lucas, J. M., Guitart, M., et al. (1996). FRAXE mutation analysis in three Spanish families. Am J Med Genet. 64, 434-440. doi:10.1002/(SICI)1096-8628(19960809)64:2<434::AID-AJMG40>3.0.CO;2-D

Farwell, K. D., Shahmirzadi, L., El-Khechen, D., Powis, Z., Chao, E. C., Tippin Davis,

B., et al. (2015). Enhanced utility of family-centered diagnostic exome sequencing with inheritance model-based analysis: results from 500 unselected families with undiagnosed genetic conditions. Genet Med. 17, 578-586. doi:10.1038/gim.2014.154

Gecz, J., Oostra, B. A., Hockey, A., Carbonell, P., Turner, G., Haan, E. A., et al. (1997). FMR2 expression in families with FRAXE mental retardation. Hum Mol Genet. 6, 435-441. doi:10.1093/hmg/6.3.435

Gedeon, A. K., Meinanen, M., Ades, L. C., Kaariainen, H., Gecz, J., Baker, E., et al. (1995). Overlapping submicroscopic deletions in Xq28 in two unrelated boys with developmental disorders: identification of a gene near FRAXE. Am J Hum Genet. 56, 907-914.

Grozeva, D., Carss, K., Spasic-Boskovic, O., Tejada, M. I., Gecz, J., Shaw, M., et al. (2015). Targeted next-generation sequencing analysis of 1,000 individuals with intellectual disability. Hum Mutat. 36, 1197-1204. doi:10.1002/humu.22901

Honda, S., Hayashi, S., Kato, M., Niida, Y., Hayasaka, K., Okuyama, T., et al. (2007). Clinical and molecular cytogenetic characterization of two patients with non-mutational aberrations of the FMR2 gene. Am J Med Genet A. 143A, 687-693. doi:10.1002/ajmg.a.31638

Isrie, M., Froyen, G., Devriendt, K., de Ravel, T., Fryns, J. P., Vermeesch, J. R., et al. (2012). Sporadic male patients with intellectual disability: contribution of X-chromosome copy number variants. Eur J Med Genet. 55, 577-585. doi:10.1016/j.ejmg.2012.05.005

Jiang, Y. H., Yuen, R. K., Jin, X., Wang, M., Chen, N., Wu, X., et al. (2013). Detection of clinically relevant genetic variants in autism spectrum disorder by whole-genome sequencing. Am J Hum Genet. 93, 249-263.

doi:10.1016/j.ajhg.2013.06.012

Knight, S. J., Flannery, A. V., Hirst, M. C., Campbell, L., Christodoulou, Z., Phelps, S. R., et al. (1993). Trinucleotide repeat amplification and hypermethylation of a CpG island in FRAXE mental retardation. Cell. 74, 127-134. doi:10.1016/0092-8674(93)90300-f

Knight, S. J., Ritchie, R. J., Chakrabarti, L., Cross, G., Taylor, G. R., Mueller, R. F., et al. (1996). A study of FRAXE in mentally retarded individuals referred for fragile X syndrome (FRAXA) testing in the United Kingdom. Am J Hum Genet. 58, 906-913.

Kosmicki, J. A., Samocha, K. E., Howrigan, D. P., Sanders, S. J., Slowikowski, K., Lek, M., et al. (2017). Refining the role of de novo protein-truncating variants in neurodevelopmental disorders by using population reference samples. Nat Genet. 49, 504-510. doi:10.1038/ng.3789

Lesca, G., Biancalana, V., Brunel, M. J., Quack, B., Calender, A., Lespinasse, J. (2003). Clinical, cytogenetic, and molecular description of a FRAXE French family. Psychiatr Genet. 13, 43-46. doi:10.1097/00041444-200303000-00007

Lim, E. T., Raychaudhuri, S., Sanders, S. J., Stevens, C., Sabo, A., MacArthur, D. G., et al. (2013). Rare complete knockouts in humans: population distribution and significant role in autism spectrum disorders. Neuron. 77, 235-242. doi:10.1016/j.neuron.2012.12.029

Lo Nigro, C., Faravelli, F., Cavani, S., Perroni, L., Novello, P., Vitali, M., et al. (2000). FRAXE mutation in a mentally retarded subject and in his phenotypically normal twin brother. Eur J Hum Genet. 8, 157-162. doi:10.1038/sj.ejhg.5200425

Mila, M., Sanchez, A., Badenas, C., Brun, C., Jimenez, D., Villa, M. P., et al. (1997).

Screening for FMR1 and FMR2 mutations in 222 individuals from Spanish special schools: identification of a case of FRAXE-associated mental retardation. Hum Genet. 100, 503-507. doi:10.1007/s004390050542

Mondal, K., Ramachandran, D., Patel, V. C., Hagen, K. R., Bose, P., Cutler, D. J., et al. (2012). Excess variants in AFF2 detected by massively parallel sequencing of males with autism spectrum disorder. Hum Mol Genet. 21, 4356-4364. doi:10.1093/hmg/dds267

Moore, S. J., Strain, L., Cole, G. F., Miedzybrodzka, Z., Kelly, K. F., Dean, J. C. (1999). Fragile X syndrome with FMR1 and FMR2 deletion. J Med Genet. 36, 565-566.

Mulley, J. C., Yu, S., Loesch, D. Z., Hay, D. A., Donnelly, A., Gedeon, A. K., et al. (1995). FRAXE and mental retardation. J Med Genet. 32, 162-169. doi:10.1136/jmg.32.3.162

RK, C. Y., Merico, D., Bookman, M., J, L. H., Thiruvahindrapuram, B., Patel, R. V., et al. (2017). Whole genome sequencing resource identifies 18 new candidate genes for autism spectrum disorder. Nat Neurosci. 20, 602-611. doi:10.1038/nn.4524

Sahoo, T., Theisen, A., Marble, M., Tervo, R., Rosenfeld, J. A., Torchia, B. S., et al. (2011). Microdeletion of Xq28 involving the AFF2 (FMR2) gene in two unrelated males with developmental delay. Am J Med Genet A. 155A, 3110-3115. doi:10.1002/ajmg.a.34345

Stettner, G. M., Shoukier, M., Hoger, C., Brockmann, K., Auber, B. (2011). Familial intellectual disability and autistic behavior caused by a small FMR2 gene deletion. Am J Med Genet A. 155A, 2003-2007. doi:10.1002/ajmg.a.34122

Timms, K. M., Bondeson, M. L., Ansari-Lari, M. A., Lagerstedt, K., Muzny, D. M., Dugan-Rocha, S. P., et al. (1997). Molecular and phenotypic variation in

patients with severe Hunter syndrome. Hum Mol Genet. 6, 479-486. doi:10.1093/hmg/6.3.479

Whibley, A. C., Plagnol, V., Tarpey, P. S., Abidi, F., Fullston, T., Choma, M. K., et al. (2010). Fine-scale survey of X chromosome copy number variants and indels underlying intellectual disability. Am J Hum Genet. 87, 173-188. doi:10.1016/j.ajhg.2010.06.017

Willemsen, M. H., de Leeuw, N., de Brouwer, A. P., Pfundt, R., Hehir-Kwa, J. Y., Yntema, H. G., et al. (2012). Interpretation of clinical relevance of X-chromosome copy number variations identified in a large cohort of individuals with cognitive disorders and/or congenital anomalies. Eur J Med Genet. 55, 586-598. doi:10.1016/j.ejmg.2012.05.001

Wolff, D. J., Gustashaw, K. M., Zurcher, V., Ko, L., White, W., Weiss, L., et al. (1997). Deletions in Xq26.3-q27.3 including FMR1 result in a severe phenotype in a male and variable phenotypes in females depending upon the X inactivation pattern. Hum Genet. 100, 256-261. doi:10.1007/s004390050501

Zhang, Y., Kong, W., Gao, Y., Liu, X., Gao, K., Xie, H., et al. (2015). Gene mutation analysis in 253 chinese children with unexplained epilepsy and intellectual/developmental disabilities. PLoS One. 10, e0141782. doi:10.1371/journal.pone.0141782
